# Development and Validation of a Stability-Indicating HPLC Method for the Simultaneous Determination of Florfenicol and Flunixin Meglumine Combination in an Injectable Solution

**DOI:** 10.1155/2017/1529280

**Published:** 2017-07-11

**Authors:** Nidal Batrawi, Hani Naseef, Fuad Al-Rimawi

**Affiliations:** ^1^Samih Darwazah Institute for Pharmaceutical Industries, Faculty of Pharmacy, Nursing and Health Professions, Birzeit University, West Bank, State of Palestine; ^2^Department of Chemistry and Chemical Technology, Faculty of Science and Technology, Al-Quds University, Jerusalem 20002, State of Palestine

## Abstract

The combination of the powerful antimicrobial agent florfenicol and the nonsteroidal anti-inflammatory flunixin meglumine is used for the treatment of bovine respiratory disease (BRD) and control of BRD-associated pyrexia, in beef and nonlactating dairy cattle. This study describes the development and validation of an HPLC-UV method for the simultaneous determination of florfenicol and flunixin, in an injectable preparation with a mixture of excipients. The proposed RP-HPLC method was developed by a reversed phase- (RP-) C18e (250 mm × 4.6 mm, 5 *μ*m) column at room temperature, with an isocratic mobile phase of acetonitrile and water mixture, and pH was adjusted to 2.8 using diluted phosphoric acid, a flow rate of 1.0 mL/min, and ultraviolet detection at 268 nm. The stability-indicating method was developed by exposing the drugs to stress conditions of acid and base hydrolysis, oxidation, photodegradation, and thermal degradation; the obtained degraded products were successfully separated from the APIs. This method was validated in accordance with FDA and ICH guidelines and showed excellent linearity, accuracy, precision, specificity, robustness, LOD, LOQ, and system suitability results within the acceptance criteria.

## 1. Introduction

Florfenicol and flunixin meglumine combination (Flr&Flx) is an effective antimicrobial and nonsteroidal anti-inflammatory for veterinary use, indicated for treatment of bovine respiratory disease (BRD) associated with* Mannheimia haemolytica*,* Pasteurella multocida*,* Histophilus somni*, and* Mycoplasma bovis*, and control of BRD-associated pyrexia in beef and nonlactating dairy cattle.

Florfenicol is a broad-spectrum, primarily bacteriostatic antibiotic, effective against wide range of pathogenic strains of microorganisms including many Gram-negative and Gram-positive bacteria [1].

**Figure 1:**
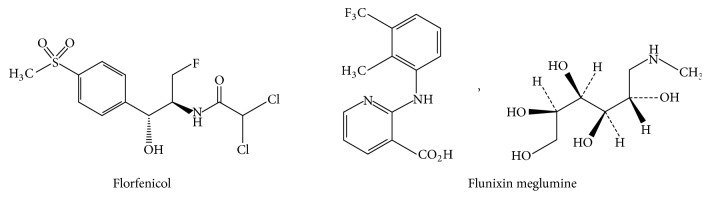
Chemical structure of florfenicol and flunixin meglumine.

Flunixin is cyclooxygenase inhibitor analgesic anti-inflammatory used to reduce the hemodynamic inflammation caused by endotoxin and to reduce mortality associated with endotoxemic shock [2, 3]. The structure of florfenicol and flunixin is shown in Figure 1.

By reviewing the literature, there are many analytical methods for individual determination of Flr or Flx or in combination with other drugs in a pharmaceutical formulation, but none of these methods include stability-indicating analytical method for the simultaneous determination of both Flr and Flx, in the presence of degradation materials [4–11]. The objective of this study is therefore to develop and validate a simple and fast RP-HPLC method using UV-PDA detector to simultaneously quantify florfenicol and flunixin in a medicinal formulation. The developed method is a validated stability-indicating method, which provides a high degree of analytical confidence that it can be used for the assay test of both active ingredients in a single run and can specifically detect any potential degradants that may produce during stability testing or during product shelf life. This method was validated in accordance with the requirements of FDA, ICH, and USP guidelines [12–16].

## 2. Materials and Methods

### 2.1. Instrumentation

Liquid chromatography method development and validation analysis were conducted using Dionex-Ultimate 3000 HPLC system, equipped with LPG-3400SD pump, WPS-3000SL autosampler, TCC-3000 column oven, DAD-3000 UV–VIS diode array detector, and Phenomenex Luna C18 (5 *μ*m × 25 cm × 4.6 mm id) column. Chromeleon Data system Software (Version 6.80 DU10A Build 2826 (171948)) was used for data processing and evaluation. The used double-distilled water was prepared by Aquatron equipment model A 4000D.

### 2.2. Chemicals and Reagents

Active materials, florfenicol and flunixin meglumine, working reference standards with a purity of (>99%) were purchased from Sigma Aldrich. The finished injectable solution samples and all active materials and excipients were gifted by the Advanced Veterinary Manufacturing Company (Palestine). The acetonitrile used was of HPLC grade and water was obtained by double distillation. Other reagents such as phosphoric acid, hydrochloric acid, sodium hydroxide, and hydrogen peroxide were purchased from Merck and Sigma Aldrich.

### 2.3. Chromatographic Conditions

Mobile phase was prepared by mixing 600 mL acetonitrile with 400 mL of water and then adjusted to pH 2.8 using 2 M phosphoric acid. The chromatographic conditions were run as shown in Table 1.

### 2.4. Preparation of Standard Solutions

A standard solution of florfenicol (1.2 mg/mL) and flunixin meglumine (0.1096 mg/mL) was prepared by dissolving an accurately weighed amount of florfenicol 300 mg and 27.4 mg of flunixin meglumine in 50 mL of mobile phase, and then 5 mL of the resulting solution was diluted to 25 mL by the same solvent.

### 2.5. Preparation of Sample Solution

A sample solution was prepared with a concentration equivalent to that in standard solution by transferring 1 mL of the drug injectable solution, which contains 300 mg of florfenicol and 27.4 mg of flunixin meglumine, with about 40 mL of the mobile phase into a 50 mL volumetric flask; the volume was completed to mark by the same solvent, and then 5 mL of the resulting solution was diluted to 25 mL by the same solvent.

### 2.6. Method Validation

The method was validated as per ICH and FDA guidelines for specificity, linearity and range, accuracy, precision, LOQ, LOD, and robustness [12, 15].

#### 2.6.1. Specificity

Forced degradation study was conducted by exposing samples of the drug substance and drug product to various stress conditions of hydrolysis, oxidation, photodegradation, and thermal stress; the time and conditions are illustrated in Table 2. Stressed samples were analyzed occasionally; related peaks were checked for the retention times, peaks interference, spectra purity, and separation factors.

#### 2.6.2. Linearity

To evaluate linearity and range of the method, seven different concentrations of florfenicol (480, 720, 960, 1200, 1440, 1680, and 1920 *μ*g/mL) and flunixin meglumine (43.8, 65.8, 87.7, 109.6, 131.5, 153.4, and 175.4 *μ*g/mL) were prepared. Three injections from each concentration were analyzed under the same conditions.

#### 2.6.3. Accuracy

The accuracy of the assay method was performed on three spiked concentration levels (80%, 100%, and 120%) around the test concentration (florfenicol 1200 *μ*g/mL and flunixin meglumine 109.6 *μ*g/mL), by nine determinations (three replicates of each concentration). The percentage recovery and RSD were calculated for each of the replicate samples.

#### 2.6.4. Precision

Precision was performed at two levels, repeatability and intermediate precision. Repeatability, or method precision, was established by six assay determinations at the 100% concentration levels on the same day. The RSD of obtained results was calculated to evaluate repeatability results.

Intermediate precision or ruggedness was established by doing repeatability test by another analyst on a different day and using different equipment. The RSD of combined results obtained by both analysts was calculated to evaluate intermediate-precision results.

#### 2.6.5. LOD and LOQ

LOD and LOQ of florfenicol and flunixin using this method were determined by analyzing different dilute solutions of florfenicol and flunixin and measuring signal-to-noise ratio. The limit of detection (LOD) is the concentration that gives a signal-to-noise ratio of approximately 3 : 1, while the limit of quantification (LOQ) is the concentration that gives a signal-to-noise ratio of approximately 10 : 1 with % RSD (*n* = 3) of less than 10%.

#### 2.6.6. Robustness

Robustness was performed by applying little deliberate changes of the following method conditions:

- pH of mobile phase: ±0.2
- Temperature: ±5°C
- Flow rate: ±0.1 mL/min
- Wavelength: ±2 nm
- Mobile phase composition, organic composition ±5%

Sample and standard solutions were analyzed for each change. Change was made to evaluate its effect on the method. Obtained data for each case was evaluated by calculating % RSD and percent of recovery.

## 3. Results and Discussion

### 3.1. Method Development and Optimization

With regard to the physical and chemical properties of the analytes and the information obtained from the literature, analytical method was developed to select a preliminary reversed phase HPLC-UV chromatographic conditions, including detection wavelength, mobile phase, stationary phase, and sample preparation procedure. For that, series of trials were performed, such as different compositions of mobile phase and different types of stationary phase and column lengths, with different pH values and buffering agents.

On the basis that the method will be used for separation of two analytes from each other, and also from their degradants, the RP18e stationary phase with a 250 mm length was initially selected. According to the analytes physicochemical properties, a mixture of acetonitrile and water 50% : 50% v/v was selected as the mobile phase, adjusted to pH 4.2 with diluted acetic acid and a flow rate of 1.0 mL/min.

Using these isocratic chromatographic conditions, first successful effort of eluting the analytes simultaneously has been established; the florfenicol peak symmetry and column efficiency were good, but the flunixin peak eluted lately with poor symmetry and column efficiency.

This required carrying out some modifications in the mobile phase composition and its pH value. Therefore, the ratio of the mobile phase components was changed to be acetonitrile and water 60% : 40% v/v and the pH reduced to 3.0 by diluted acetic acid.

Good flunixin peak symmetry and column efficiency were obtained, but the florfenicol peak was affected.

**Figure 2:**
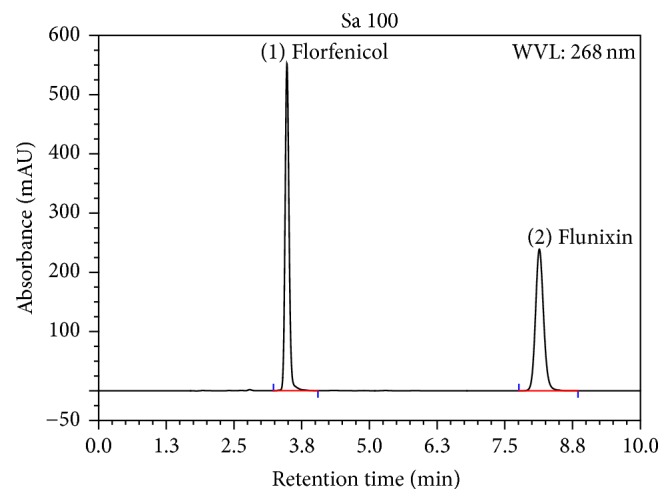
Chromatogram of Flr and Flx in drug product using the developed method in this study.

Additional chromatographic conditions were altered to optimize the florfenicol peak, where the pH of the same mobile phase was reduced to 2.8 by diluted phosphoric acid. As a result of that, a satisfying analytical method was obtained as shown in Figure 2, the resolution (*R*) and other system suitability parameters of the obtained peaks of florfenicol and flunixin were excellent, as illustrated in Table 7.

**Figure 3:**
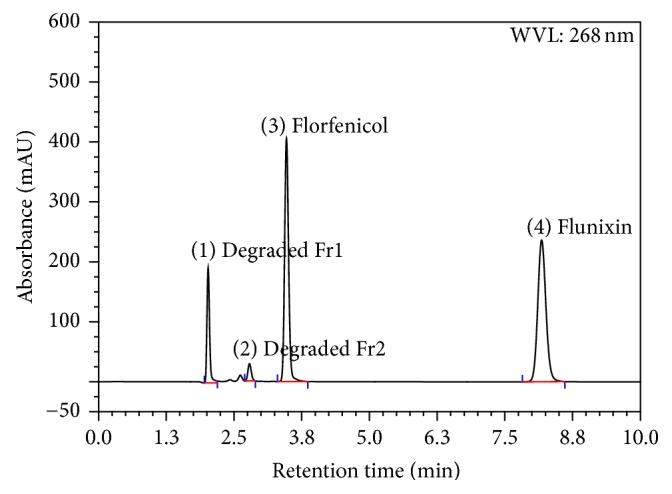
Chromatogram of stress testing of Flr and Flx under basic hydrolysis condition of 0.02 N NaOH, at RT for 2 hours.

**Figure 4:**
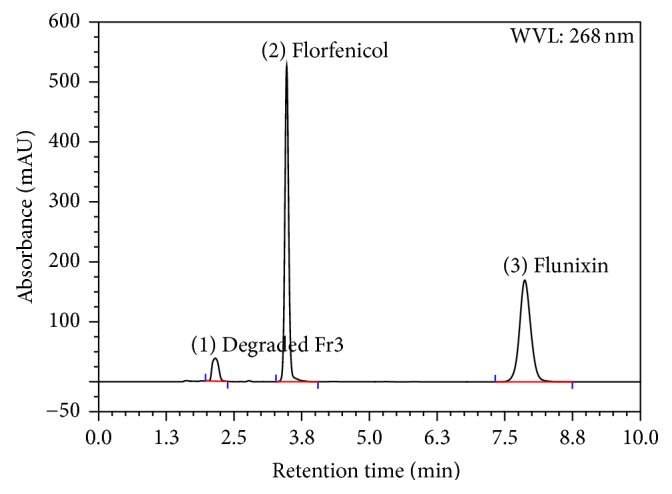
Chromatogram of stress testing of Flr and Flx under acidic hydrolysis condition of 1 N HCl, at 40°C for 2 days.

**Figure 5:**
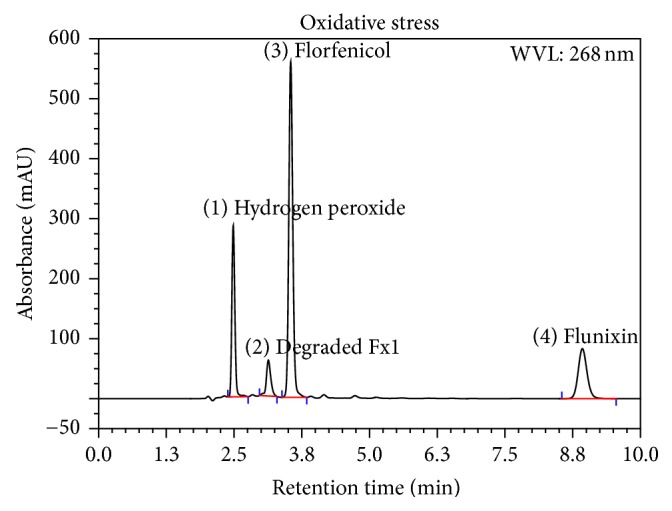
Chromatogram of stress testing of Flr and Flx under oxidative condition of 0.2% H_2_O_2_ at 40°C, protected from light for 7 days.

**Figure 6:**
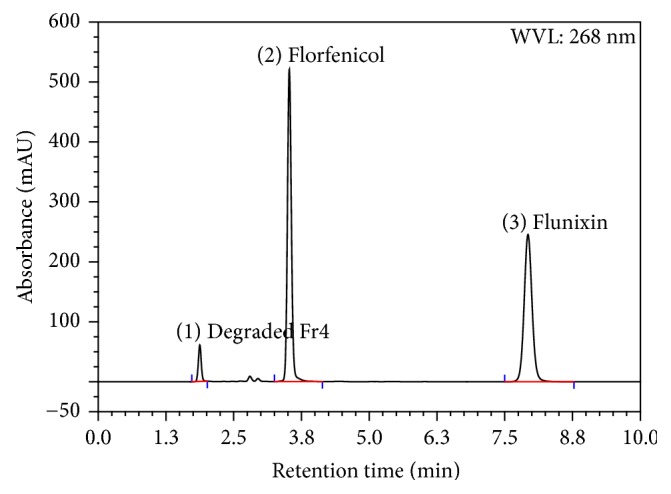
Chromatogram of Flr and Flx under thermal stress testing condition of 75°C for 14 days.

Using the PDA-UV a WL of 268 nm was selected as the optimum wavelength. Placebo (mixture of excipients) did not show any response. Forced degradation study solutions were analyzed using the developed method and the degradative materials peaks were adequately separated from that of Flr and Flx (Figures 3, 4, 5, and 6). The optimized conditions were given in Table 1.

### 3.2. Specificity and Stability-Indicating Study

Specificity is the ability of the analytical method to measure the active ingredient response in the presence of other excipients and its potential degradants. Forced degradation was carried out to evaluate the specificity and stability-indicating properties of the method, by exposing samples of the drug substance and drug product to stress conditions of hydrolysis, oxidation, photodegradation, and thermal degradation as detailed under Section 2.6.1.

Stress testing of the drug product was performed to induce force degradation and determine degradation pathways and help evaluate the stability of the drug substance and also validate specificity of the analytical procedures.

The basic condition applied on the active drug substances for 2 hours induced the hydrolysis of florfenicol causing assay loss of about 26% and degradative materials (Fr1) and (Fr2) of about 23% and 4.5%, respectively, while no degradation was observed for flunixin.

The acidic condition applied on the active drug substances for 2 days induced the hydrolysis of florfenicol causing assay loss of about 10.5% and degradative material (Fr3) of about 11%, while no degradation was observed for flunixin.

The oxidative condition applied on the active drug substances for 7 days induced the oxidation of flunixin causing assay loss of about 61% and degradative material (Fx1) of about 14.5%, while no degradation was observed for florfenicol.

The thermal condition applied on the active drug substances for 14 days induced the degradation of florfenicol causing assay loss of about 7.5% and degradative material (Fr4) of about 8%, while no degradation was observed for flunixin.

There was no evidence of degradation of the drug product exposed to stress condition of the photodegradation type. These results are summarized in Table 3.

Results showed no interference between the chromatographic peaks of florfenicol and flunixin and the excipients, impurities, and degradation products under the various stress conditions (Figures 3, 4, 5, and 6). The spectra of all the peaks were checked using PDA showing perfect purity.

It is concluded that method of analysis is qualified and reliable to demonstrate and detect any expected change in the drug product assay during stability studies.

### 3.3. Linearity and Range

The linearity of an analytical method can be defined as the ability of the method to obtain test results that are directly proportional to the analyte concentration, within a given range. The linearity of the method was observed in the concentration range of 480 *μ*g/mL to 1920 *μ*g/mL for florfenicol and 43.8 *μ*g/mL to 175.4 *μ*g/mL for flunixin demonstrating its suitability for analysis. The goodness of fit (*R*^2^) was found to be 0.9997 for each of Flr and Flx, respectively, indicating a linear relationship between the concentration of analyte and area under the peak, as shown in Table 4.

### 3.4. Accuracy

The accuracy of an analytical procedure expresses the closeness of results obtained by that method to the true value. The results of accuracy testing showed that the method is accurate within the acceptable limits. The percentage recovery and RSD were calculated for both active ingredients florfenicol and flunixin; all the results are within limits. Acceptable accuracy was within the range of 98.0% to 102.0% recovery and not more than 2.0% RSD, as demonstrated in Table 5.

### 3.5. Precision

Precision of an analytical method is defined as “the closeness of agreement between a series of measurements obtained from multiple sampling of the same homogeneous sample under the prescribed conditions,” and it is normally expressed as the relative standard deviation.

The results of repeatability and intermediate-precision testing showed that the method is precise within the acceptable limits. The RSD were calculated for both active ingredients florfenicol and flunixin; all the results are within limits. Precision was not more than 2.0% RSD, as demonstrated in Table 6.

### 3.6. Robustness

The robustness of the method was examined using the minor modifications, as shown in Section 2.6.6. The results of robustness testing showed that little change of method conditions, such as pH of the mobile phase, composition of the mobile phase, temperature, flow rate, and wavelength, does not affect the method significantly, and so it is robust within the acceptable limits. Percent of recovery was within the range of 97.0% to 103.0% and RSD was not more than 3.0% for both active ingredients, florfenicol and flunixin.

### 3.7. Limit of Detection and Limit of Quantification (LOD and LOQ)

The limit of detection (LOD) is the lowest amount of analyte in a sample that can be detected, but not necessarily quantitated, while the limit of quantification (LOQ) is the lowest amount of analyte in a sample that can be quantitatively determined with suitable precision. The method showed a LOD of 0.60 and 0.20 *μ*g/mL for florfenicol and flunixin, respectively, and showed a LOQ of 2.4 and 0.40 *μ*g/mL for florfenicol and flunixin, respectively, with a RSD (*n* = 3) of 2.4% and 2.6% for florfenicol and flunixin, respectively.

### 3.8. System Suitability

System suitability parameters were performed using six replicates of a standard solution containing both florfenicol and flunixin, to verify the analytical system performance. The method shows that the % RSD values are not more than 2.0% for both florfenicol and flunixin, and all the values for the system suitability parameters such as the column efficiency, the tailing factors, and the resolution values, as presented in Table 7, are within limits.

### 3.9. Solution Stability

The stability of solutions was performed at room temperature, by the assay analysis at regular intervals. The solution was tested every 2 hours from the beginning to 16 hours. The percent of recovery was within the range of 98.0% to 102.0% and RSD was not more than 2.0% for both active ingredients, florfenicol and flunixin, indicating a good stability of sample and standard solutions for 16 hrs.

## 4. Conclusion

A fast, simple, accurate, precise, and linear stability-indicating HPLC method has been developed and validated for the simultaneous analysis of florfenicol and flunixin in a pharmaceutical formulation. The method is stability indicating and reliable to detect and quantify any potential degradation in the drug product during stability studies and can be used for routine quality control analysis. The method is robust enough to reproduce accurate and precise results under different chromatographic conditions.

## Figures and Tables

**Table 1:** HPLC chromatographic conditions of the current method.

Chromatographic conditions	
Flow rate	1.0 mL/min
Wavelength (*λ*)	268 nm
Stationary phase	RP18e, 5 *µ*m, 250 × 4.6 mm
Column temperature	25°C
Injection volume	20 *µ*L
Run time	10 minutes

**Table 2:** Stress conditions applied for drug substance and drug product.

Stress type	Conditions	Time
Acid hydrolysis	1 N HCl; at 40°C	2 days
Base hydrolysis	0.02 N NaOH; at RT	2 hours
Oxidative/solution	0.2% H_2_O_2_ at 40°C; protected from light	7 days
Thermal	75°C	14 days
Photodegradation	UV light	3 days

**Table 3:** The results of stress testing of Flr and Flx under various conditions.

Stress type	Detectable change	Degradation	
		Name	Percentage
Basic hydrolysis	26% florfenicol assay loss Degradation	Fr1 Fr2	23.0% 4.5%
Acid hydrolysis	10.5% florfenicol assay loss Degradation	Fr3	11.0%
Oxidative/solution	61% flunixin assay loss Degradation	Fx1	14.5%
Thermal	7.5% florfenicol assay loss Degradation	Fr4	8.0%
Photodegradation	No change		

**Table 4:** Regression analysis of florfenicol and flunixin.

API	Linearity range (*µ*g/mL)	(*R*^2^)	Linearity equation	*Y*-intercept
Flr	480 to 1920	0.9997	*y* = 0.0395*x* + 0.1003	0.10
Flx	43.8 to 175.4	0.9997	*y* = 0.3795*x* + 0.1361	0.13

**Table 5:** Evaluation of the accuracy of the method developed in this study.

API	Spiked level (*µ*g/mL)	Replicate number	Recovery (*µ*g/mL)	% mean recovery	% RSD
Flr	960.0	1	960.79	99.84	0.22
		2	957.58		
		3	956.90		
	1200.0	1	1214.99	101.10	0.15
		2	1213.30		
		3	1211.35		
	1440.0	1	1431.03	99.43	0.07
		2	1432.83		
		3	1431.33		

Flx	87.7	1	87.59	99.62	0.23
		2	87.21		
		3	87.29		
	109.6	1	110.72	100.83	0.18
		2	110.49		
		3	110.33		
	131.5	1	130.55	99.31	0.03
		2	130.62		
		3	130.60		

**Table 6:** Evaluation of precision of the method developed in this study.

API	Flr	Flx
Spiked amount (*µ*g/mL)	1200	109.6
Intermediate precision (ruggedness)		
Replicate number	Recovery (*µ*g/mL)	
Repeatability (method precision)		
	Day 1	
1	1214.99	110.72
2	1213.30	110.49
3	1211.35	110.33
4	1210.77	110.26
5	1209.64	110.09
6	1208.75	110.10

Mean recovery	1211.47	110.33
% RSD	0.19	0.22

	Day 2	
1	1204.72	110.29
2	1198.82	113.07
3	1200.00	110.95
4	1202.35	110.27
5	1200.59	111.19
6	1202.35	108.61

Mean recovery	1206.47	110.53
% RSD	0.47	0.92

**Table 7:** System suitability parameters of the current method.

	Florfenicol	Flunixin	Acceptance criteria
Tailing factor, *T*	1.10	1.12	≤2.0
Resolution, *R*	4.4		>2.0
Number of theoretical plates, *N*	11500	14700	>2000
% RSD (*n* = 6)	0.19	0.22	≤2.0%

## References

[1] United States Pharmacopeial Convention Vet. Syst. Florfenicol.

[2] British Pharmacopoeia (2013). Monograph on Flunixin meglumine. *British Pharmacopoeia*.

[3] United States Pharmacopeial Convention Vet. Syst. Flunixin.

[4] Nasim A., Aslam B., Javed I. (2016). Determination of florfenicol residues in broiler meat and liver samples using RP-HPLC with UV-visible detection. *Journal of the Science of Food and Agriculture*.

[5] Orlando E. A., Costa Roque A. G., Losekann M. E., Colnaghi Simionato A. V. (2016). UPLC–MS/MS determination of florfenicol and florfenicol amine antimicrobial residues in tilapia muscle. *Journal of Chromatography B: Analytical Technologies in the Biomedical and Life Sciences*.

[6] Guo L., Tian X., Shan S., Han J., Shang X., Ma S. (2014). Simultaneous determination of florfenicol and diclazuril in compound powder by RP-HPLC-UV method. *Journal of Chemistry*.

[7] Karami-Osboo R., Miri R., Javidnia K., Kobarfard F. (2016). Simultaneous chloramphenicol and florfenicol determination by a validated DLLME-HPLC-UV method in pasteurized milk. *Iranian Journal of Pharmaceutical Research*.

[8] Song J.-S., Park S.-J., Choi J.-Y. (2016). Development of analytical method and monitoring of veterinary drug residues in Korean animal products. *Korean Journal for Food Science of Animal Resources*.

[9] Meucci V., Vanni M., Sgorbini M., Odore R., Minunni M., Intorre L. (2013). Determination of phenylbutazone and flunixin meglumine in equine plasma by electrochemical-based sensing coupled to selective extraction with molecularly imprinted polymers. *Sensors and Actuators, B: Chemical*.

[10] Belal F. F., Abd El-Razeq S. A., Fouad M. M., Fouad F. A. (2015). Micellar high performance liquid chromatographic determination of flunixin meglumine in bulk, pharmaceutical dosage forms, bovine liver and kidney. *Analytical Chemistry Research*.

[11] Jedziniak P., Szprengier-Juszkiewicz T., Olejnik M., Jaroszewski J. (2007). Determination of flunixin and 5-hydroxyflunixin in bovine plasma with HPLC-UV method development, validation and verification. *Bulletin of the Veterinary Institute in Pulawy*.

[12] International Conference on Harmonisation of Technical Requirements for Registration of Pharmaceuticals for Human Use ICH Q2 (R1) Validation of Analytical Procedures: Text and Methodology.

[13] Pharmacopeia U. S. (2009). USP-NF <1225> Validation of Compendial Methods. *USP 32-NF27*.

[14] (2003). *Stability Testing of New Drug Substances and Products*.

[15] FDA (2014). *Guidance for Industry Analytical Procedures and Methods Validation for Drugs and Biologics*.

[16] Batrawi N., Wahdan S., Al-Rimawi F. (2017). A validated stability-indicating hplc method for simultaneous determination of amoxicillin and enrofloxacin combination in an injectable suspension. *Scientia Pharmaceutica*.

